# Translational Pharmacokinetic/Pharmacodynamic Modeling and Simulation of Oxaliplatin and Irinotecan in Colorectal Cancer

**DOI:** 10.3390/pharmaceutics15092274

**Published:** 2023-09-03

**Authors:** Jinwei Zhu, Yicui Zhang, Yixin Zhao, Jingwei Zhang, Kun Hao, Hua He

**Affiliations:** 1 State Key Laboratory of Natural Medicines, Jiangsu Province Key Laboratory of Drug Metabolism and Pharmacokinetics, Institute of Pharmaceutical Sciences, China Pharmaceutical University, Nanjing 210009, China; 2 Center of Drug Metabolism and Pharmacokinetics, School of Pharmacy, China Pharmaceutical University, Nanjing 210009, China

**Keywords:** translational pharmacokinetic/pharmacodynamic (PK/PD) modeling, oxaliplatin, irinotecan, colorectal cancer

## Abstract

Despite the recent advances in this field, there are limited methods for translating organoid-based study results to clinical response. The goal of this study was to develop a pharmacokinetic/pharmacodynamic (PK/PD) model to facilitate the translation, using oxaliplatin and irinotecan treatments with colorectal cancer (CRC) as examples. The PK models were developed using qualified oxaliplatin and irinotecan PK data from the literature. The PD models were developed based on antitumor efficacy data of SN-38 and oxaliplatin evaluated in vitro using tumor organoids. To predict the clinical response, translational scaling of the models was established by incorporating predicted ultrafiltration platinum in plasma or SN-38 in tumors to PD models as the driver of efficacy. The final PK/PD model can predict PK profiles and responses following treatments with oxaliplatin or irinotecan. After generation of virtual patient cohorts, this model simulated their tumor shrinkages following treatments, which were used in analyzing the efficacies of the two treatments. Consistent with the published clinical trials, the model simulation suggested similar patient responses following the treatments of oxaliplatin and irinotecan with regards to the probabilities of progression-free survival (HR = 1.05, 95%CI [0.97;1.15]) and the objective response rate (OR = 1.15, 95%CI [1.00;1.32]). This proposed translational PK/PD modeling approach provides a significant tool for predicting clinical responses of different agents, which may help decision-making in drug development and guide clinical trial design.

## 1. Introduction

Cancer drug development continues to have a high fail rate, at 95%, in clinical trials, with a lack of efficacy being the leading cause of 60% of all unsuccessful outcomes [1]. The development of reliable preclinical models has been considered the foundation for screening effective drug candidates [2]. In addition, a successful translational approach is essential in applying these models to predict clinical efficacy for candidate screening and dosing schedule optimization [2,3].

Tumor organoids (PDTOs) are complex 3D tissues that are cultured from tumor fragments. PDTOs contain multiple cell types and can reproduce some key anatomical and functional characteristics of human tumors [4,5]. Unlike traditional cell culture technologies, PDTOs can recapitulate the tumor microenvironment and mimic a variety of cell–cell and cell–extracellular matrix interactions. In addition, they are capable of preserving the histopathological and main clinical genetic features of parental tumors [6,7]. Thus, PDTOs have been considered a more human-relevant in vitro model for evaluating the efficacy of antitumor agents. By now, PDTO-based in vitro studies have been widely verified and used in antitumor drug screening and precision medicine. Helen Yan et al. established a living biobank with 46 gastric cancer organoid lines representing different molecular subtypes. Through drug testing, two PDTOs exhibited a favorable response to both cisplatin and 5-flurouracil, consistent with the patients’ responses after treatment with cisplatin and 5-flurouracil. Conversely, another PDTO, obtained from a patient with progressive disease following 5-flurouracial-based treatment demonstrated a negative response to 5-flurouracil. These results suggested the effectiveness of PDTO in predicting drug sensitivity. This living biobank was further utilized to assess the sensitivity of gastric cancer organoids to marketed drugs or drug candidates, identifying abemaciclib as a potential drug [8].

In addition, PDTOs have also been applied to identify responders to specific drugs [9,10,11]. In the case of oxaliplatin and irinotecan, first-line treatments for metastatic colorectal cancer (CRC), these drugs were co-cultured with tumor organoids derived from CRC patients. The growth of tumor organoids derived from responders was significantly inhibited by oxaliplatin and SN-38 (the active metabolite of irinotecan), suggesting a good in vitro–in vivo correlation between PDTO response and clinical response. Therefore, PDTOs have been considered a promising tool for classifying the responders to oxaliplatin and irinotecan in CRC patients [6,9,12].

Beyond their qualitative applications in drug screening and responder identification, a particularly attractive application of a PDTO-based in vitro study is the quantitative prediction of the clinical responses of antitumor drugs. This approach has the potential to optimize the drug combination and dosing schedule in drug development. Compared to static culture conditions in vitro, the time-dependent nature of drug exposure in vivo poses a challenge in in vitro to in vivo translation (IVIVT). However, drug exposure in tumor tissue is a time-dependent variable in vivo, which is a major barrier in in vitro to in vivo translation (IVIVT). The pharmacokinetic/pharmacodynamic (PK/PD) model is a valuable approach to establishing the relationship between drug dose, exposure, and response [13,14]. Through the integration of knowledge of physiology, disease processes, and pharmacology, various mathematical models have been established to optimize doses, design clinical trials, and provide efficacy evidence [13,15].

Given the association between PDTO results and the clinical responses of patients [16], an in vitro PD model based on PDTO efficacy data can be used to predict clinical response. Model-based virtual clinical trials, also known as in silico clinical simulations, leverage a virtual patient cohort and mathematical models to predict the potential clinical benefits of drugs and drug candidates [17,18]. It also can identify responder groups for treatment, explore drug combinations, predict real clinical trials, and more [19].

The key aspect of conducting a model-based virtual clinical trial is to generate a virtual patient population that can replicate the characteristics and variability of clinical patients [20]. For example, Aman Singh et al. developed a PK/PD model using clinical information from multiple myeloma patients to generate virtual patients and perform a model-based virtual clinical trial simulation to support dose selection for clinical trial design [21]. Similarly, a quantitative system pharmacology (QSP) platform for immune oncology has also been implemented in multiple virtual clinical trial simulations where a subset of parameters was selected to generate a virtual patient cohort resembling the clinical patients. Tumor shrinkages and biomarker changes of these virtual patient cohorts were simulated in order to provide prospective information for clinical trials [22,23]. As a promising tool for drug development, model-based virtual clinical trial simulation can further explore treatment benefits based on PDTO experiments.

In the current research, PK/PD models have been developed with in vitro PDTO data, and model-based virtual clinical trials have been simulated to assess the benefits of oxaliplatin and/or irinotecan treatment in CRC patients. Model-based virtual clinical trials successfully predicted the antitumor efficacy of these two drugs, demonstrating good consistency with previous clinical practice. This study provided a translational approach to assessing the clinical response of new drug candidates or therapies based on PDTO efficacy data.

## 2. Materials and Methods

### 2.1. Data Collection of Pharmacokinetics and Pharmacodynamics

PK data came from previous studies. PubMed was used to collect oxaliplatin and irinotecan PK data from mice and humans. The keywords used in the search were (‘oxaliplatin’ OR ‘irinotecan’) AND (‘pharmacokinetic’) AND (‘mice’ OR ‘human’). The inclusion and exclusion criteria were as follows: (1) the administration approach was either intravenous bolus or infusion of antitumor drug solution; (2) no other drug was simultaneously administered in the preclinical study; (3) studies that focused on specific populations were excluded, such as those with hepatic or renal impairment; and (4) considering that the blood concentration varies from plasma concentration, studies that only reported blood concentrations were excluded. The plasma and/or tumor concentration time profiles were converted to numerical data using the GetData Graph Digitizer (GetData Pty Ltd., Kogarah, Australia). PD data of the drug-sensitivity test in tumor organoids was gifted by Accurate International Biotechnology (Guangzhou, China) to establish the PK/PD model. The experimental conditions were described in a previous study [24]. In brief, organoids underwent treatment with a range of drug concentrations. After a 96-h incubation period, tumor organoids were mixed with 100 μL of CellTiter-Glo 3D reagent for a 5-min interval, followed by an additional 25-min incubation. Subsequently, cell viability was determined by luminescence measurements, which were normalized to those of vehicle controls.

### 2.2. Modeling of In Vitro Pharmacodynamic and In Vivo Plasma Pharmacokinetics in Mice and Humans

The natural growth of PDTOs in the current study was described using an exponential growth model. Hill’s function was employed to describe the inhibitory effect of drugs on PDTO growth. The differential Equations (1)–(3) of the PD model are as follows:

(1) $$\frac{{dV}_{t\_c}}{dt}=k_{g}*V_{t\_c}$$

(2) $$\frac{{dV}_{t\_t}}{dt}=k_{g}*V_{t\_t}-\left(\frac{E_{max}*C^{hill}}{{EC}_{50}^{hill}+C^{hill}}\right)*V_{t\_t}$$

(3) $$Cell\;viability=\frac{V_{t\_t}}{V_{t\_c}}\;$$

where k_g_ is the natural growth rate of organoids and V_t_c_ and V_t_t_ are PDTO volumes. E_max, EC_50_, and hill are used to describe the killing effects of antitumor drugs.

Generally, the PK studies for oxaliplatin were based on ultrafiltration platinum in plasma instead of oxaliplatin molecules, which were considered to be perfused into tumors as active forms. Therefore, a two-compartment model was used to describe the behavior of ultrafiltration platinum in plasma. The differential Equations (4) and (5) used are as follows:

(4) $$V_{C\_OXA}*\frac{{dC}_{C\_OXA}}{dt}=-\left({CL}_{OXA}+Q_{OXA}\right)*C_{C\_OXA}+Q_{OXA}*C_{P\_OXA}\;$$

(5) $$V_{P\_OXA}*\frac{{dC}_{P\_OXA}}{dt}=Q_{OXA}*\left(C_{C\_OXA}-C_{P\_OXA}\right)$$

where the C_C_OXA_ and C_P_OXA_ represent the concentrations of ultrafiltration platinum in the central compartment and peripheral compartment. Q_OXA_ is the plasma flow rate in the peripheral compartment. V_C_OXA_ and V_P_OXA_ are the central and peripheral compartment volumes, respectively. CL_OXA_ is the clearance of ultrafiltration platinum.

For irinotecan and its active metabolite SN-38, a minimal PBPK (mPBPK) model was developed. Tissues other than tumor were collectively grouped as “other” compartments. The tumor compartment was divided into two sub-compartments, vascular and tissue, while other organs and tissues were grouped as the “other” compartment. The differential Equations (6)–(12) are as follows:

(6) $$V_{C\_IRI}*\frac{{dC}_{C\_IRI}}{dt}=-\left({CL}_{IRI}+Q_{IRI}+Q_{t}\right)*C_{C\_IRI}+Q_{IRI}*C_{O\_IRI}+Q_{t}*C_{TIS\_IRI}\;$$

(7) $$V_{O\_IRI}*\frac{{dC}_{O\_IRI}}{dt}=Q_{IRI}*\left(C_{C\_IRI}-C_{O\_IRI}\right)$$

(8) $$V_{TIS\_IRI}*\frac{{dC}_{TIS\_IRI}}{dt}=Q_{t}*\left(C_{C\_IRI}*{fu}_{IRI}-C_{TIS\_IRI}\right)-{PS}_{IRI}*(C_{TIS\_IRI}-\frac{C_{TC\_IRI}}{K_{P\_IRI}})\;$$

(9) $$V_{TC\_IRI}*\frac{{dC}_{TC\_IRI}}{dt}={PS}_{IRI}*(C_{TIS\_IRI}-\frac{C_{TC\_IRI}}{K_{P\_IRI}})\;$$

(10) $$V_{C\_SN}*\frac{{dC}_{C\_SN}}{dt}={CL}_{M\_SN}*C_{C\_IRI}*\frac{V_{C\_IRI}}{V_{C\_SN}}+Q_{SN}*C_{O\_SN}+Q_{t}*\frac{C_{T\_SN}}{K_{P\_SN}}-\left({CL}_{SN}+Q_{SN}+Q_{t}\right)*C_{C\_SN}\;$$

(11) $$V_{O\_SN}*\frac{{dC}_{O\_SN}}{dt}=Q_{SN}*\left(C_{C\_SN}-C_{O\_SN}\right)\;$$

(12) $$V_{T}*\frac{{dC}_{T\_SN}}{dt}=Q_{t}*\left(C_{C\_SN}*{fu}_{SN}-\frac{C_{T\_SN}}{K_{P\_SN}}\right)\;$$

Here, C_C_IRI_, C_O_IRI_, and C_T_IRI_ represent the concentration of irinotecan in the central compartment, “other” compartment, and tumor compartment. C_TIS_IRI_ and C_TC_IRI_ are the concentrations of irinotecan in the tumor interstitial space and tumor cells, respectively. C_C_SN_, C_O_SN_, and C_T_SN_ are the concentrations of SN-38 in the central compartment, “other” compartment, and tumor compartment. Q_IRI_ and Q_SN_ are the plasma flow rates in the “other” compartment. V_C_IRI_, V_C_SN_, V_O_IRI_, and V_O_SN_ are the central compartment and other compartment volumes. CL_IRI_ and CL_SN_ are the clearance of irinotecan and SN-38. CL_M_SN_ is the metabolic rate of irinotecan in SN-38. K_P_IRI_ and K_P_SN_ are tumor/plasma partition coefficients. PS_IRI_ is the permeation rate of irinotecan entering into the tumor cells in interstitial space.

**Figure 1:**
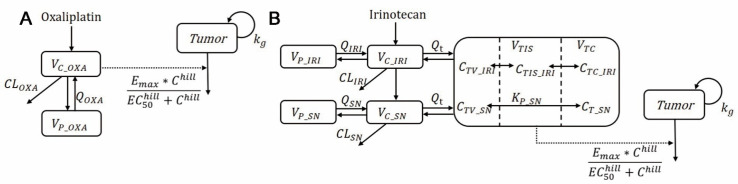
The schematic structure of the PK-PD models for oxaliplatin (**A**) and irinotecan (**B**). V_C_OXA_, V_P_OXA_, V_C_IRI_, V_P_IRI_, V_C_SN_, V_P_SN_: apparent volume of distribution in central and peripheral compartment; V_TIS_, V_TC_: tumor volume; Q_OXA_, Q_IRI_, Q_SN_: clearance between central and peripheral compartments; Q_t_: blood flow rate between central and tumor compartments; CL_OXA_, CL_IRI_, CL_SN_: systematic clearance; K_P_SN_: tumor/plasma partition coefficient; C_TV_IRI_, C_TIS_IRI_, C_TC_IRI_, C_TV_SN_, C_T_SN_: drug concentration in tumor compartments; K_g_: tumor growth rate; E_max_: the maximum killing effect; EC_50_: drug concentration of half maximum effect; hill: Hill efficient.

The mPBPK model for irinotecan was first developed based on the PK data of tumor-bearing mice. To further develop the mPBPK model for humans, some tumor-related parameters from the mouse model were scaled up and integrated with other parameters based on human PK data. The mimicked ultrafiltration platinum in plasma and SN-38 in tumor tissue was considered a driver of efficacy. To describe tumor growth in vivo, the Gompertz model replaced the exponential growth model because of its growth limitation. Figure 1 shows the global structures of the final PK/PD model. All models were built using Monolix 2019R2 (Simulations Plus, Orsay, France) with the stochastic approximation expectation-maximization (SAEM) method.

### 2.3. Simulation of Tumor Shrinkage in Humans

Simulations were conducted using Berkeley Madonna (version 10.2.6; UC Berkeley, Berkeley, CA, USA). Different dosing regimens were employed to simulate the tumor growth profiles of various patients based on the final PK/PD models. To make the simulation more clinically relevant, the two most common dosages of oxaliplatin and irinotecan were simulated and are listed in Table 1. The efficacy of the treatments was assessed according to the Response Evaluation Criteria In Solid Tumors (RECIST 1.1) [25].

### 2.4. Model-Based Virtual Clinical Trial

In the model-based virtual clinical trial, a virtual patient cohort was generated according to the pathological baseline of CRC patients with 30% variation by Monte Carlo sampling. Specific parameters related to the drug effect were selected to represent the interindividual variabilities of the drug effect. The mean value was the geometric mean of the in vitro PD model parameters. Monte Carlo sampling was used to allocated values for these selected parameters. The overall predicted response rate of virtual patients based on RECIST 1.1 was divided into three situations: progressive disease, stable disease, and partial or complete response. To assess the two treatments, the probabilities of progression-free survival (PFS) and objective response rate (ORR) were analyzed and plotted.

### 2.5. Statistical Analysis

All statistical analyses were based on R (version 4.0.2; R Foundation for Statistical Computing, Vienna, Austria) and RStudio (version 1.3.1037; R Foundation for Statistical Computing, Vienna, Austria), and the Kaplan-Meier plotter was performed using survival packages. Hazard ratios (HRs) and 95% confidence intervals were calculated and used to compare the PFS of the two treatment groups. ORR was compared by odds ratios (ORs).

## 3. Results

### 3.1. Development of an In Vitro PD Model

**Figure 2:**
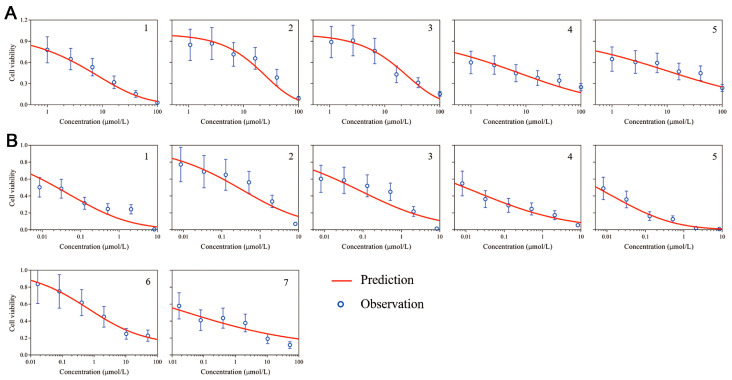
Individual fitting plot of the in vitro PD model for 96 h treatment with oxaliplatin (**A**) or SN-38 (**B**). 1–7 are the numbers of samples provided drug test data.

The PD data from the in vitro PDTO provided by Accurate International Biotechnology (Guangzhou, China) was used to develop the PD model. The Hill model was developed to describe the concentration–effect relationship of PDTOs in response to oxaliplatin and SN-38. The estimated patient-specific parameters of the PD model are exhibited in Table 2. The growth rate of PDTO was fixed at 0.03, according to the natural growth of the PDTOs. For oxaliplatin, the estimate values of EC50 ranged from 246 μmol/L to 622 μmol/L across the patients. This indicates that different PDTOs showed varying sensitivities to oxaliplatin. Similarly, the estimated values of EC50 for SN-38 ranged from 4.17 μmol/L to 15.5 μmol/L. Hill coefficients in the oxaliplatin PD model ranged from 0.384–0.977 and showed a less steep profile, with a narrower range (0.2–0.435) in the SN-38 PD model. As shown in Figure 2, these estimated individual parameters effectively captured the relationship between drug concentration and cell viability.

### 3.2. Development of the Oxaliplatin and Irinotecan PK Model

**Figure 3:**
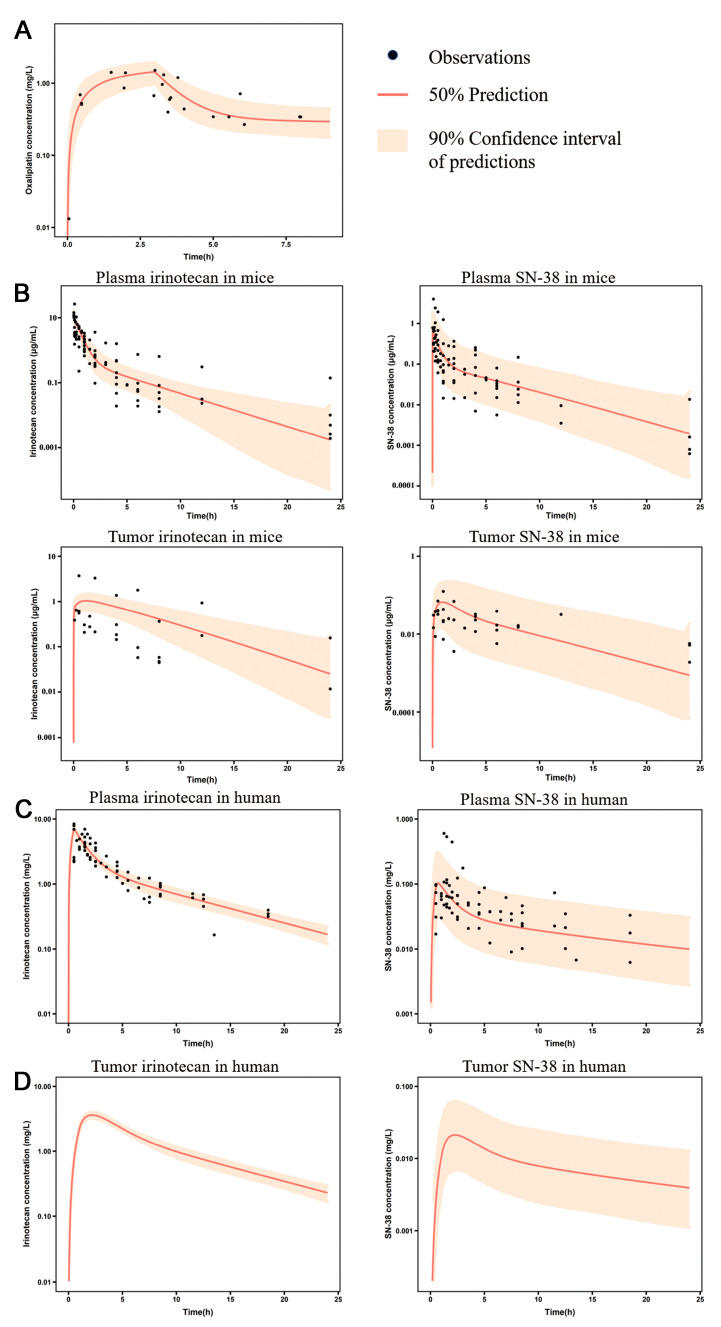
Visual predictive check (VPC) plots of PK models for oxaliplatin and irinotecan. (**A**) VPC plot of a PK model for oxaliplatin in human. (**B**) VPC plots of a minimal PBPK model for irinotecan in a tumor-bearing mouse. (**C**) VPC plots of a PK model for irinotecan in humans. (**D**) Simulated irinotecan and SN-38 profiles in human tumor tissue.

**Figure A1:**
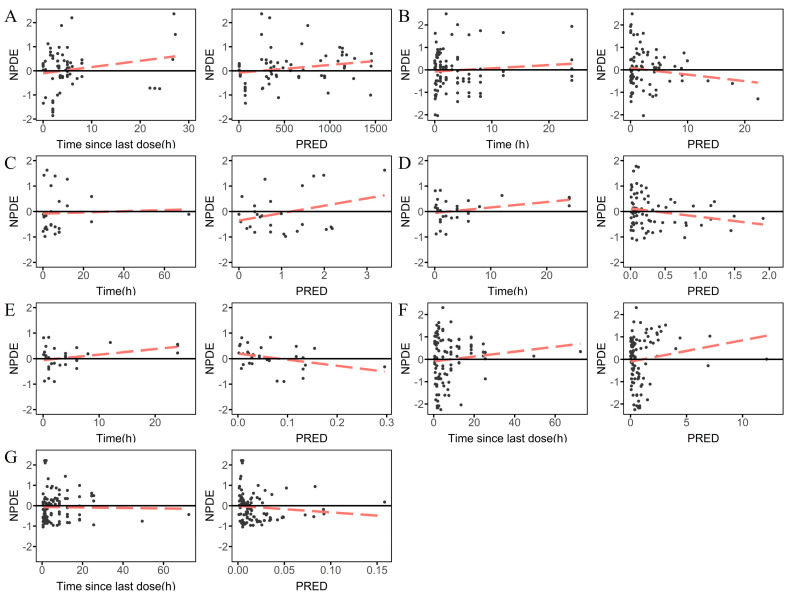
Plots of normalized prediction distribution errors (NPDE) versus time and predictions for pharmacokinetic (PK) models. (**A**). NPDE versus time and population prediction (PRED) for the oxaliplatin PK model. (**B**). NPDE versus time and PRED for irinotecan concentration in mice plasma. (**C**). NPDE versus time and PRED for irinotecan concentration in mice tumors. (**D**). NPDE versus time and PRED for SN-38 concentration in mice plasma. (**E**). NPDE versus time and PRED for SN-38 concentration in mice tumors. (**F**). NPDE versus time and PRED for irinotecan concentration in human plasma. (**G**). NPDE versus time and PRED for SN-38 concentration in human plasma.

Two different PK models were developed to describe the PK behavior of oxaliplatin and irinotecan. For oxaliplatin, the unbound plasma drug concentration was considered the PD driver to describe the antitumor effect. A two-compartmental PK model was developed to describe the disposition of ultrafiltration platinum in the body, in which the parameters were estimated using the plasma PK data from human studies. For irinotecan, the antitumor effect of irinotecan was determined by the disposition of SN-38 in the tumor interstitial fluid, which was described using an mPBPK model. In this model, a two-compartmental PK model was introduced to describe the disposition of irinotecan. SN-38 was generated from irinotecan metabolism, and its disposition was also described using a two-compartmental PK model. In addition, a tumor compartment with blood vessel, interstitial fluid, and cell sub-compartments was incorporated to describe exposure to irinotecan and SN-38. Plasma PK data from humans were used to estimate a portion of the PK parameters for irinotecan and SN-38. Due to the absence of drug concentrations in human tumors, plasma and tumor concentration profiles of both irinotecan and SN-38 from tumor-bearing mice were collected and fitted to obtain the drug disposition parameters in tumors. Besides PS_IRI_, most parameters were estimated with good precision. In preclinical to clinical translation, physiological parameters, such as tumor volumes and blood flow rate in tumors, were fixed with species-dependent values, while drug-related parameters, such as tumor/plasma partition coefficients and permeation rate, were scaled according to tumor volumes. The PK parameters of oxaliplatin and irinotecan are summarized in Table 3, and the visual predictive check (VPC) with 1000 simulations is shown in Figure 3. The VPC results suggested that these PK models had the ability to reproduce drug profiles and simulate their distribution in tumors (Figure 3). The plots of normalized prediction distribution errors (NPDE) versus time and population predictions are shown in Appendix Figure A1. Most NPDE were evenly distributed around zero against PRED and time (or time since last dose).

### 3.3. The Combination of the PK/PD Model and the Simulation of Tumor Shrinkage

**Figure 4:**
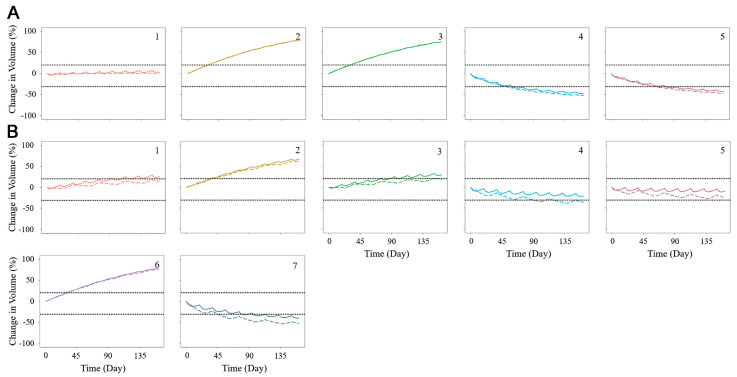
Individual predictions of tumor shrinkage with oxaliplatin (**A**) and irinotecan (**B**) treatment. The solid line represents the diameter change with the maximum tolerated dose and the dash line is the diameter change with a low dose and high dosage-frequency treatment. 1–7 are the numbers of samples provided drug test data.

To develop the PK/PD model of the antitumor effects of oxaliplatin and irinotecan, the simulated unbound platinum and tumor interstitial SN-38 concentrations were incorporated into the PD model. The final PK/PD model could simulate the tumor size changes following the treatments with oxaliplatin or irinotecan. As shown in Figure 4, tumor shrinkage profiles were predicted successfully for different drugs. These clinical outcomes were categorized according to RECIST 1.1, which defines partial response (30% decrease), progressive disease (20% increase), and stable disease. These categories are marked in Figure 4 to help identify the potential response. Compared with irinotecan, oxaliplatin might be more effective for Patient 1, as the predicted result showed that this patient might achieve a stable disease state with oxaliplatin treatment but a progression disease state with irinotecan treatment. Patient 2 could not benefit from either oxaliplatin or irinotecan therapies, and Patient 3 preferred irinotecan over oxaliplatin. Patients 4 and 5 could benefit from both treatments, with oxaliplatin being slightly more effective than irinotecan. Patient 6 had progressive disease regardless of the treatment. Patient 7 had a partial response after irinotecan treatment.

### 3.4. Model-Based Virtual Clinical Trial

**Figure 5:**
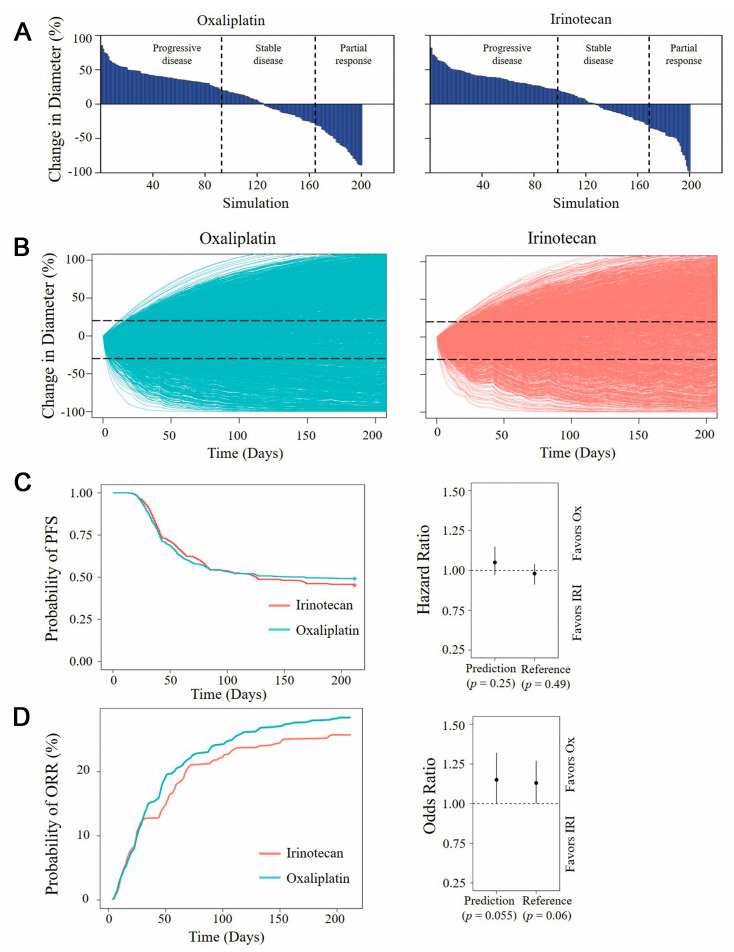
Virtual clinical trial simulation results. (**A**) The percentage change in tumor diameter after 12-week treatments. (**B**) Tumor growth profiles of 2000 virtual patients with different treatments. (**C**) The probability of PFS of oxaliplatin and irinotecan treatment (left) and hazard ratio from prediction and clinical practice (right). (**D**) The probability of ORR of oxaliplatin and irinotecan treatment and odds ratio from prediction and clinical practice (right). PFS progression free survival, ORR objective response rate.

Based on the value and distribution of parameters in in vitro PD models, a Monte Carlo simulation was conducted to compare the clinical antitumor benefits of oxaliplatin and irinotecan. The parameters used in sampling are listed in Supporting Information Table 4. The percentage change in tumor diameter (*n* = 200) at 12 weeks was evaluated using waterfall plots (Figure 5A). With oxaliplatin treatment, 46.5%of the virtual patients had a progressive disease state, 35% had a stable disease state, and 18.5% had a partial/complete response. Irinotecan therapy demonstrated similar benefits to oxaliplatin following 12-week treatment. The ratio of virtual patients with partial responses decreased from 18.5% to 16%, and the ratio of progression diseases increased from 46.5% to 49%.

Further model-based virtual clinical trials were set up to simulate the tumor shrinkage of 2000 virtual patients with different treatments (Figure 5B). As shown in Figure 5C,D, the probabilities of PFS and ORR of the two drugs were statistically analyzed. The results did not show significant differences between the two agents. The HR for PFS suggested that oxaliplatin treatment might improve PFS compared with irinotecan, but there was no significant difference (HR = 1.05, 95%CI [0.97;1.15], *p* = 0.25). The OR for ORR also indicated that oxaliplatin had a slight benefit in terms of ORR, but the difference was not significant (OR = 1.15, 95%CI [1.00;1.32], *p* = 0.055).These results were consistent with previous clinical trials, which reported similar clinical benefits between oxaliplatin-based and irinotecan-based regimens [29].The irinotecan-based regimen slightly improved PFS (HR = 0.98, 95%CI [0.91;1.04], *p* = 0.49), while the oxaliplatin-based regimen showed better ORR (OR = 1.13, 95%CI [1.00;1.27], *p* = 0.06).

## 4. Discussion

Considering the significant correlations between PDTO response and clinical outcomes, tumor organoids can be a potential in vitro tool for drug candidate screening [9,16]. Generally, the in vitro concentration–effect relationship of antitumor agents can be estimated using PDTOs, with IC50 commonly utilized as the metric for candidate screening [30]. However, this study emphasizes that tumor exposure of the candidates is also a critical factor in determining in vivo efficacy. The achievement of the expected antitumor efficacy relies on the desired drug exposure in tumors. The PK/PD model is an ideal tool that integrates information on exposure and effects to predict the clinical efficacy of drug candidates [31,32,33,34].

In the current study, a PK/PD model-based IVIVT approach was employed to predict the clinical benefits of oxaliplatin and irinotecan based on in vitro PDTO data. The antitumor effect was first determined using PDTOs and then used to develop a PD model. Patient-specific parameters were estimated due to variations in PDTO response and data limitations. The range of EC50 values also represents the significantly different efficacy of the drugs, with oxaliplatin requiring higher concentrations for a similar inhibition effect compared to SN-38.

In vitro, the medium drug concentration is commonly considered the driver of efficacy to quantify the exposure–response relationship. In IVIVT, tumor interstitial drug exposure is considered a surrogate to drive efficacy in vivo. Therefore, the prediction of interstitial drug exposure is a crucial process for translating the in vitro effect to in vivo efficacy. For irinotecan, it is first metabolized to the active metabolite SN-38, which is then distributed to tumor tissue. An mPBPK model with a tumor compartment was developed to describe the distribution of irinotecan and SN-38 in tumors. Due to the absence of tumor exposure data in irinotecan and SN-38, preclinical data from mice were utilized to estimate tumor distribution-related parameters, which were then translated into the human mPBPK model to predict drug exposure in human tumor interstitial fluid [35]. For oxaliplatin, there is no tumor distribution data available, so ultrafiltration platinum exposure is commonly considered identical in plasma and interstitial fluids. In the current study, PK models described plasma ultrafiltration platinum and tumor interstitial SN-38 concentrations separately, which were utilized as the drivers in the PD models to predict their antitumor efficacy.

Model-based virtual clinical trials have been applied to predict clinical efficacy, to identify predictive biomarkers, to facilitate patient selection, and to improve clinical trial design in antitumor drug development [21,22,23,36]. Generally, virtual patients are generated to simulate the characteristics and variability of real clinical patients, forming the basis for model-based virtual clinical trials. PK/PD models were implemented to simulate the responses of patients, such as tumor shrinkage. All the simulation data could be used for statistical analysis of drug efficacy, for identifying predictive biomarkers, and for determining suitable patient populations. For instance, a model-based virtual clinical trial simulation based on patients with hepatocellular carcinoma was conducted to explore the clinical benefits of nivolumab and ipilimumab combined treatments and to determine predictive biomarkers that could facilitate patient selection [36]. In the current study, virtual patients were generated using Monte Carlo sampling based on the value and distribution of the model parameters. To avoid being influenced by extremes, the geometric means of individual parameters were calculated as the median of sampling [37]. Based on these virtual patients, model-based virtual clinical trials were applied to evaluate the clinical benefits of oxaliplatin or irinotecan. The dosing regimens in the simulations were designed according to drug specifications and classic regimens [38,39]. The predicted clinical response was then compared to the real-world data from clinical trials to validate the developed IVIVT approach.

According to the results of the model-based virtual clinical trial, oxaliplatin slightly improved ORR (*p* = 0.055), with an OR of 1.15 (95% CI, 1.00–1.32). Similarly, the HR for PFS was 1.05 (95% CI, 0.97–1.15), indicating no significant difference (*p* = 0.025) between oxaliplatin and irinotecan treatments. These trends align with a previous meta-analysis [29]. This mentioned research integrated 19 clinical trials involving 4571 patients to compare irinotecan and oxaliplatin as first-line therapies for metastatic CRC. According to real clinical practice, there was no significant difference in ORR (OR = 1.13, 95%CI [1.00;1.27], *p* = 0.06) or PFS (HR = 0.98, 95%CI [0.91;1.04], *p* = 0.49) between oxaliplatin and irinotecan treatments. The consistency between real clinical trials and model-based virtual clinical trials demonstrates the utility of PDTO-based translational approaches in predicting the clinical responses of drug candidates or therapies compared to currently used treatments.

Notably, this approach aims to evaluate the potential clinical responses of drug candidates and therapies in virtual patients through comparison with marketed drugs, rather than providing precise predictions of PFS or ORR. In addition, this current study only attempted monotherapy simulations, and more research on combination therapies should be conducted to better align with clinical reality. While the overall analysis did not reveal a significant difference in the efficacy of irinotecan-based and oxaliplatin-based regimens, there were discernible variations when oxaliplatin and irinotecan were employed in combination [29]. For instance, when coupled with 5-flurouracil, irinotecan and oxaliplatin have shown comparable clinical benefits. However, the combination of anti-VEGF antibody and irinotecan demonstrated enhanced PFS. To increase the applicability of this approach, models for combination treatment will be the primary objective of forthcoming studies. Considering the possible interactions of combined drugs, more in vitro studies are required to support these analyses [40,41]. Moreover, this study lacks individual validation. The reliability of individual simulations of tumor shrinkage may require more validation.

In summary, this study, based on PDTO data, developed PD models for oxaliplatin and irinotecan and integrated them with PK models to establish a PK/PD model that described their dose–exposure–response relationship. By generating virtual patient cohorts and conducting a set of model-based virtual clinical trials, this study predicted the clinical efficacy of oxaliplatin and irinotecan treatments accurately. This approach shows the promise in predicting the clinical efficacy of antitumor drugs and has the strong potential to be applied in antitumor drug candidates or therapies, which can help decision-making in drug development.

## 5. Conclusions

This study successfully developed a PDTO-based translational PK/PD model incorporating in vitro PDTO data, animal PK data, and clinical PK data to predict tumor shrinkage following oxaliplatin and irinotecan treatments accurately. The further model-based virtual clinical trial demonstrated comparable clinical efficacy between the two drugs, aligning with previous clinical practice. These findings manifest the strong potential of this translational approach to assess the clinical efficacy of antitumor agents or other candidates. Overall, this study provides an IVIVT approach to successfully predict the potential clinical responses of antitumor agents or candidates.

## Figures and Tables

**Table 1:** Designed dosing regimens of oxaliplatin and irinotecan.

Drug	Title 2	Title 3
Irinotecan	350 mg/m^2^	Once every 3 weeks
	125 mg/m^2^	Once a week for 4 consecutive weeks, followed by a two-week rest period
Oxaliplatin	130 mg/m^2^	Once every 3 weeks
	85 mg/m^2^	Once every 2 weeks

**Table 2:** The individual parameters of pharmacodynamic model.

Patient No.		Oxaliplatin				SN-38	
	kg	E_max_	EC_50_	Hill	E_max_	EC_50_	Hill
1	0.03	0.095	308	0.614	0.076	15.50	0.349
2		0.095	246	0.977	0.040	12.40	0.396
3		0.105	352	0.891	0.047	10.70	0.324
4		0.056	622	0.397	0.049	7.04	0.266
5		0.038	354	0.384	0.103	15.50	0.325
6		-	-	-	0.023	6.10	0.435
7		-	-	-	0.027	4.17	0.200

**Table 3:** Parameter estimates of the PK models for oxaliplatin and irinotecan.

Parameters	Definition	Unit	Mouse	IIV (RSE%)	Human	IIV (RSE%)	Sources
			Estimates (RSE%)		Estimates (RSE%)		
V_C_OXA_	Apparent volumes of distribution in the central compartment of oxaliplatin	L	-	-	49.9 (46.3)	1.05 (30.9)	Estimated
V_P_OXA_	Apparent volumes of distribution in the peripheral compartment of oxaliplatin	L	-	-	538 (29.3)	-	Estimated
CL_OXA_	Systematic clearance of oxaliplatin	L/h	-	-	5.96 (42.5)	0.597 (63.6)	Estimated
Q_OXA_	Clearance between central and peripheral compartments of oxaliplatin	L/h	-	-	49.3 (29.7)	-	Estimated
V_C_IRI_	Apparent volumes of distribution in the central compartment of irinotecan	L	0.0349 (32)	0.873 (20.4)	72.1 (6.78)	1.62 (39.3)	Estimated
V_P_IRI_	Apparent volumes of distribution in the peripheral compartment of irinotecan	L	0.0493 (25.8)	-	93.4 (15.2)	-	Estimated
V_TIS_	Volumes of tumor interstitial space	mL	0.1 (fixed)	-	2 (fixed)	-	Assumed
V_TC_	Volumes of tumor cells	mL	0.4 (fixed)	-	8 (fixed)	-	Assumed
V_T_	Volumes of tumor	mL	0.5 (fixed)	-	10 (fixed)	-	[26]
V_C_SN_	Apparent volumes of distribution in the central compartment of SN-38	L	0.00122 (15.9)	0.494 (59.3)	11.2 (34.5)	0.139 (71.9)	Estimated
V_P_SN_	Apparent volumes of distribution in the peripheral compartment of SN-38	L	0.108 (33.1)	-	706 (52.7)	-	Estimated
CL_IRI_	Systematic clearance of irinotecan	L/h	0.0527 (19.7)	0.627 (20.4)	22.8 (5.69)	0.149 (27.4)	Estimated
CL_M_SN_	Metabolic rate from irinotecan to SN-38	L/h	1.65 × 10^−4^ (93.8)	0.851 (35.7)	0.216 (51.9)	0.666 (29)	Estimated
CL_SN_	Systematic clearance of SN-38	L/h	0.0402 (19.8)	0.234 (78.9)	42.8 (32.5)	0.5 (50.6)	Estimated
Q_IRI_	Clearance between central and peripheral compartments of irinotecan	L/h	0.0156 (40.8)	0.732 (26)	24.6 (28.8)	0.681 (35.8)	Estimated
Q_SN_	Clearance between central and peripheral compartments of SN-38	L/h	0.0369 (38.2)	0.606 (34.3)	43.5 (30.7)	0.478 (32.4)	Estimated
Q_T_	Clearance between central and tumor compartments	L/h	3.38 × 10^−3^ (fixed)	-	0.06 (fixed)	-	[26,27]
PS_IRI_	Permeation rate of irinotecan in tumor cells	cm^3^/h	0.448 (>100)	1.9 (36.5)	52 (fixed)	-	Estimated in mice/scaled in human
K_P_IRI_	Tumor/plasma partition coefficient of irinotecan	-	3.43 (74.4)	1.33 (37.1)	3.43 (fixed)	-	Estimated/constant in spieces
K_P_SN_	Tumor/plasma partition coefficient of SN-38	-	7.32 (71.6)	1.64 (30)	7.32 (fixed)	-	Estimated/constant in spieces
fu_IRI_	Fraction unbound of irinotecan in plasma	-	0.35 (fixed)	-	0.35 (fixed)	-	[28]
fu_SN_	Fraction unbound of SN-38 in plasma	-	0.05 (fixed)	-	0.05 (fixed)	-	[28]

IIV: Interindividual variability.

**Table 4:** Parameter range used to generate the Monte Carlo sampling in virtual clinical trial simulation.

Parameter	Description	Unit	Mean Value	Variable Range (%)
K_g_	Tumor growth rate	h^−1^	0.367 × 10^−3^	30
E_max_SN_	The maximum killing effect of SN-38	-	0.046	30
EC_50_SN_	SN-38 concentration of half maximum effect	μmol/L	9.2	30
hill__SN_	Hill efficient of SN-38	-	0.32	30
E_max_OXA_	The maximum killing effect of oxaliplatin	-	0.073	30
EC_50_OXA_	Oxaliplatin concentration of half maximum effect	μmol/L	358	30
hill__OXA_	Hill efficient of oxaliplatin	-	0.61	30

## Data Availability

Primary model source code and data can be obtained from the authors upon request.
